# VinCaP: a phase II trial of vinflunine in locally advanced and metastatic squamous carcinoma of the penis

**DOI:** 10.1038/s41416-021-01574-9

**Published:** 2021-10-20

**Authors:** Steve Nicholson, Holly Tovey, Tony Elliott, Stephanie M. Burnett, Clare Cruickshank, Amit Bahl, Peter Kirkbride, Anita V. Mitra, Alastair H. Thomson, Naveen Vasudev, Balaji Venugopal, Rachel Slade, Lucy Tregellas, Bruno Morgan, Alison Hassall, Emma Hall, Lisa M. Pickering

**Affiliations:** 1 Mid & South Essex NHS Foundation Trust, Essex, UK; 2 Clinical Trials and Statistics Unit, The Institute of Cancer Research, London, UK; 3 The Christie Hospital NHS Foundation Trust, Manchester, UK; 4 University Hospitals Bristol NHS Foundation Trust, Bristol, UK; 5 Clatterbridge Cancer Centre, Wirral, Merseyside UK; 6 University College London Hospitals NHS Foundation Trust, London, UK; 7 Royal Cornwall Hospital, Truro, UK; 8 St. James’s University Hospital, Leeds, UK; 9 Beatson West of Scotland Cancer Centre, Glasgow, UK; 10 University Hospitals of Leicester NHS Trust, Leicester, UK; 11 St. Georges University Hospitals Foundation Trust and The Royal Marsden Foundation Trust, London, UK

**Keywords:** Penile cancer, Penile cancer, Chemotherapy

## Abstract

**Background:**

We investigated the first-line activity of vinflunine in patients with penis cancer. Cisplatin-based combinations are commonly used, but survival is not prolonged; many patients are unfit for such treatment or experience toxicity that outweighs clinical benefit.

**Methods:**

Twenty-five patients with inoperable squamous carcinoma of the penis were recruited to a single-arm, Fleming–A’Hern exact phase II trial. Treatment comprised 4 cycles of vinflunine 320 mg/m^2^, given every 21 days. Primary endpoint was clinical benefit rate (CBR: objective responses plus stable disease) assessed after 4 cycles. Seven or more objective responses or disease stabilisations observed in 22 evaluable participants would exclude a CBR of <15%, with a true CBR of >40% being probable.

**Results:**

Twenty-two participants were evaluable. Ten objective responses or disease stabilisations were confirmed. CBR was 45.5%, meeting the primary endpoint; partial response rate was 27.3%. Seven patients received >4 cycles of vinflunine. Dose reduction or treatment delay was required for 20% of cycles. In all, 68% of patients experienced at least one grade 3 adverse event. Two deaths on treatment were not caused by disease progression.

**Conclusions:**

Pre-specified clinical activity threshold was exceeded. Toxicity was in keeping with experience in other tumours. Vinflunine merits further study in this disease.

**Trial registration:**

NCT02057913.

## Background

Platinum-based combination chemotherapy has been a conventional treatment of penis cancer for 25 years [1–3]. Objective response rates to platinum–fluoropyrimidine regimens are of the order of 30% [4] but with no clear survival benefit. Objective response rates to older regimens containing bleomycin [5], methotrexate [6], and vinca alkaloids [7, 8] were 20–30%, and often short-lived. Taxane–platinum regimens show higher response rates but with greater toxicity than these older regimens. Docetaxel, cisplatin, and 5-fluorouracil (5FU) (TPF) had an objective response rate of 38.5%, although 2/3 of patients experienced at least one grade 3/4 adverse event [9]. Pagliaro et al. [10] showed higher response rates for paclitaxel, ifosfamide, and cisplatin (TIP) in the neoadjuvant setting, giving an objective response rate of 50% in men with node-positive disease. Twenty-two patients (73.3% of the trial population) underwent subsequent surgery; three showed pathological complete remission. Grade 3/4 adverse events were seen, but TIP was deliverable in this group and is an appropriate neoadjuvant therapy where it might render resection feasible: it has not been investigated in the setting of advanced disease. Elderly patients with metastatic disease need a regimen that offers disease control with manageable toxicity.

Vinca alkaloids have been used for penis cancer since the mid-1970s, although evidence for single-agent activity is lacking [7]. Toxicity, particularly neuropathy and constipation, is dose-limiting. We investigated the activity and tolerability of vinflunine, a third-generation vinca alkaloid whose toxicity profile was expected to be more tolerable for (predominantly elderly) patients with advanced squamous penile carcinoma.

## Methods

### Eligibility criteria

Histologically proven squamous carcinoma of the penis was required, with either distant metastatic (stage M1) or locally advanced disease, defined as: any T stage with either N2 (involvement of multiple or bilateral inguinal nodes) or N3 (involvement of deep inguinal or pelvic nodes) lymph node involvement; or a T4 tumour with any N stage. Patients without distant metastases were eligible if specialist multidisciplinary team review concluded that they were unsuitable for both curative surgery and standard combination chemotherapy with the TIP regimen. Thus, such patients were only included *after* confirmation that comorbidity and/or performance status (PS) excluded a standard approach.

Participants required Eastern Cooperative Oncology Group (ECOG) PS of 0, 1, or 2 and were ineligible if they had serum concentrations of liver enzymes >2.5 times the upper limit of normal (5 times upper limit of normal in the presence of liver metastases); total bilirubin >1.5 times the upper limit of normal; calculated glomerular filtration rate (GFR) <60 mL/min; or if they had previously received any systemic chemotherapy for carcinoma of the penis. Recruitment of any patient of ECOG PS2 triggered an embargo on recruitment of PS2 patients for 4 weeks, with recommencement of PS2 enrolment subject to safety review by the Independent Data Monitoring Committee (IDMC). Previous radiotherapy was permitted, provided that this was to non-target lesions. All participants provided written informed consent.

### Treatment

Treatment comprised four 21-day cycles of vinflunine 320 mg/m^2^ given on day 1 (in 100 ml of sodium chloride 0.9% or glucose 5%) via intravenous infusion over 20 min. Treatment was repeated if neutrophil count was >1000/L and platelet count was >100,000/L. Use of prophylactic granulocyte colony-stimulating factor was permitted. Anti-emetics were given according to local policy.

Participants who had undergone prior pelvic radiotherapy and those of PS2 received their first cycle at a dose of 280 mg/m^2^, with subsequent doses being escalated to 320 mg/m^2^ in the absence of grade 3 or grade 4 haematological toxicity.

Participants must have had three bowel movements in the week prior to treatment or a bowel movement in the 48 h prior to dosing, with treatment withheld where neither of these was met. Laxatives and dietary measures to maintain bowel function were recommended from days 1 to 14 of each cycle.

Imaging comprised computed tomography of chest, abdomen, and pelvis, with magnetic resonance imaging of pelvis/penile remnant where disease was present in the penile remnant at initiation of chemotherapy. Imaging was performed within 4 weeks of the first dose of vinflunine and no later than 28 days after the fourth dose. Response Evaluation Criteria in Solid Tumours (RECIST) criteria, version 1.1 [11] was used, with independent central review. Toxicity was recorded using National Cancer Institute Common Terminology Criteria for Adverse Events (CTCAE) version 4.0 [12].

### Statistical considerations

Clinical benefit rate (CBR) was chosen as the primary endpoint, defined as the proportion of patients having achieved partial response, complete response, or stable disease according to RECIST after 4 cycles (11–12 weeks from date of first treatment). All scans were reviewed centrally by an independent radiologist. Secondary endpoints included objective response rate, toxicity, progression-free survival (time from trial entry to disease progression or death from any cause), and overall survival (time from trial entry to death from any cause).

It was assumed that CBR of <15% would be too small to warrant future trials but that a rate of ≥40% would warrant further investigation. A Fleming–A’Hern exact single-stage phase II design was used [13] (*α* = 0.05, *β* = 0.80, p0 = 0.15, and p1 = 0.40). This required 22 evaluable participants, with ≥7 objective responses or disease stabilisations required to exclude a clinical benefit of <15% (with the true rate likely to be ≥40%). No interim analyses were planned.

Patients were non-evaluable if they received no vinflunine, with replacement of patients permitted to ensure an evaluable sample size of 22 participants.

No subgroup analyses were proposed due to the small sample size. Primary and secondary endpoints are presented in the evaluable population. Sensitivity analysis for objective response rate was conducted in the measurable population (those who had RECIST assessment after completing 4 cycles or who discontinued before cycle 4 due to disease progression or death from penile cancer). Survival analyses included all participants.

CBR and objective response rate were calculated with corresponding 2-sided 95% confidence intervals. The 2-sided 90% confidence interval for CBR is also presented, equivalent to a 1-sided 95% confidence interval as per the sample size calculation. Progression-free and overall survival were analysed using Kaplan–Meier methods. Analyses (based on data as at November 20, 2017) were performed using STATA version 13.

### Study governance

The study is registered (NCT02057913), sponsored by The Institute of Cancer Research, approved by the Medicines and Healthcare Products Regulatory Authority and London Riverside Research Ethics Committee (13/LO/0822), and overseen by Independent Trial Steering and Data Monitoring Committees.

## Results

### Patient characteristics

Twenty-five patients were enrolled between June 2014 and May 2017. Participants’ characteristics are listed in Table 1 and Supplementary Table 1. Fifteen participants had distant metastases, the remaining ten having local recurrence in the penile remnant and/or pelvic lymphadenopathy.

**Table 1:** Baseline patient and tumour characteristics of all the patients entered.

		Total (*n* = 25)	
		*n*	%
Age	Mean (SD) Median (IQR) Min–Max	65.0 (10.8) 67.9 (60.4–70.4) 33.1–84.4	
Age group	<40	1	4.0
	40–50	2	8.0
	50–60	3	12.0
	60–70	10	40.0
	70–80	8	32.0
	80+	1	4.0
Ethnicity	British	23	92.0
	Irish	1	4.0
	Asian	1	4.0
Time since primary diagnosis (months)	Median (IQR)	4.9 (2.1–12.7)	
Time since primary diagnosis	<3 months	10	40.0
	3–6 months	5	20.0
	6–9 months	2	8.0
	9–12 months	1	4.0
	12 months+	7	28.0
ECOG performance status at trial entry	0	11	44.0
	1	11	44.0
	2	3	12.0
Was disease at entry the first presentation?^a^	Yes	7	28.0
	No	18	72.0
Disease stage at trial entry	3b	1	4.0
	4	24	96.0
Is residual disease present in the penis/penile remnant?	Yes	7	28.0
	No	18	72.0
TNM stage at trial entry	T1b, N3, M0	1	4.0
	T3, N3, M1	1	4.0
	T4, N0, M0	1	4.0
	T0, N0, M1	1	4.0
	T0, N2, M1	1	4.0
	T0, N3, M1	3	12.0
	T0, N3, M0	1	4.0
	T1a, N3, M1	1	4.0
	T4, N3, M0	1	4.0
	T4, N3, M1	1	4.0
	Tx, N0, M1	2	8.0
	Tx, N2, M0	1	4.0
	Tx, N2, M1	2	8.0
	Tx, N3, M0	4	16.0
	Tx, N3, M1	3	12.0
	Tx, Nx, M1	1	4.0
Prior treatment	Surgery only	16	64.0
	Surgery and radiotherapy	4	16.0
	Surgery and other^b^	1	4.0
	None	4	16.0

Loco-regional is defined as disease at the primary site and/or the local lymph node basin.

*SD* standard deviation, *IQR* interquartile range.

^a^That is, patient entered the trial for their primary disease (includes patients who were metastatic at diagnosis or who received surgery for primary disease but had residual loco-regional disease following surgery).

^b^Debridement of scrotal tumour (*n* = 1).

### Treatment

Three patients received no study treatment and are not evaluable according to the statistical analysis plan. Twenty-two participants received at least one dose of vinflunine, and 12 (55%) completed at least 4 cycles. Seven of these received >4 cycles: one participant receiving a 5th cycle, and two each receiving a total of 6, 7, and 8 cycles. Dose reduction or delay was required for 20% of doses, with the most common reason being due to adverse events (44%; 8/18).

Early discontinuations were due to adverse events/toxicity (*n* = 3; 14%), disease progression (*n* = 5; 23%), and death on treatment (*n* = 2; 9%). The adverse events leading to discontinuation were syndrome of inappropriate antidiuretic hormone secretion and pulmonary embolus (*n* = 1); constipation (*n* = 1); and neutropenia, pyrexia, and acute kidney injury (*n* = 1).

### Outcomes

Ten evaluable participants showed objective response or disease stabilisation (objective response = 6; disease stabilisation = 4), exceeding the activity threshold of 7 responses/stabilisations. CBR in the evaluable population was 45.5% (10/22; 95% confidence interval (CI): 24.4–67.8%; 90% CI: 27.1–64.7%). Objective response rate was 27.3% (6/22; 95% CI: 10.7–50.2%). All responses were partial.

**Fig. 1:**
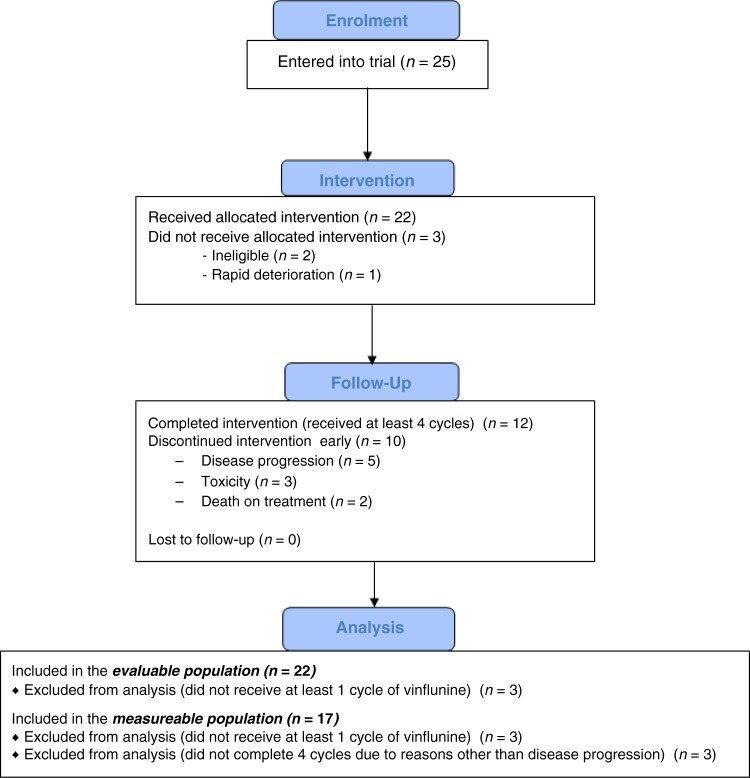
Consort diagram showing patient flow through the trial and the number of patients included in the analysis populations.

**Fig. 2:**
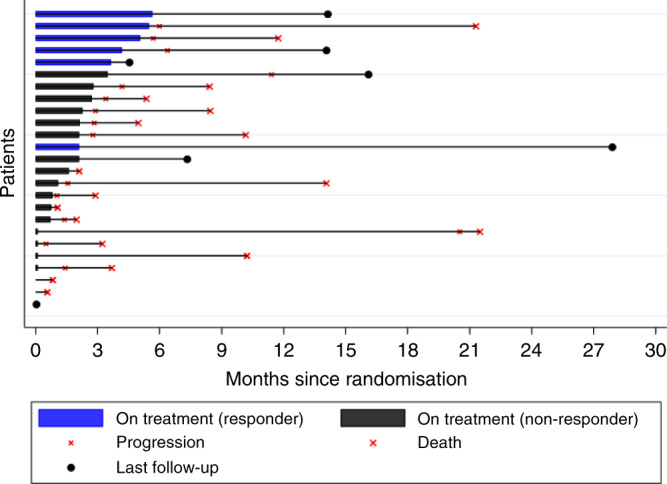
Treatment duration showing timing of treatment, progression and death for all patients by responder status.

Five participants did not complete four cycles of treatment for reasons other than disease progression (Figs. 1 and 2). Seventeen participants had measurable disease: the objective response rate for this group was 35.3% (6/17; 95% CI: 14.2–61.7%).

All participants had discontinued vinflunine at the time of analysis at median follow-up of 16.1 months (interquartile range: 14.1–27.9 months). Eighteen participants (81.8% of the evaluable population; 72% of the total recruited) had died at the time of reporting, with cause of death reported as penile cancer for 15, treatment related for 2, and 1 unrelated to cancer or treatment.

**Fig. 3:**
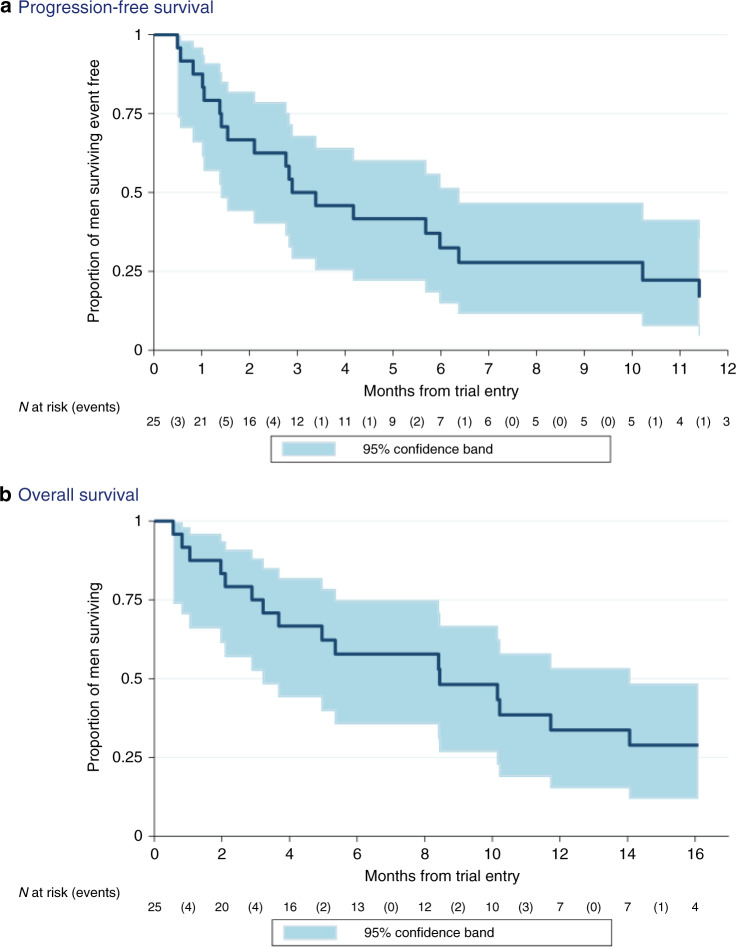
Kaplan–Meier survival curves. **a** progression-free survival (PFS) **b** overall survival (OS). Both curves are shown with associated 95% confidence bands estimated in the intention-to-treat population. N at risk (events) shows the number of patients who remain in each analysis set at a given time point and the number of PFS/OS events reported between times.

Median overall survival was 8.4 months (95% CI: 3.2–14.1 months); median progression-free survival was 2.9 months (95% CI: 1.4–6.4 months) (Fig. 3). Twelve-month overall survival was 33.7% (95% CI: 15.4–53.1%). Twelve-month progression-free survival was 16.7% (95% CI: 4.6–35.3%).

Two participants died while receiving vinflunine. Recruitment was suspended between September and November 2016 while the IDMC reviewed these incidents. These cases are presented here in outline.

Participant 1: age 66 years, ECOG PS1. He suffered CTCAE grade 5 acute kidney injury and grade 5 neutropenic sepsis commencing day 9 of cycle 2 of vinflunine. Grade 3 lethargy was the earliest reported toxicity prior to this event. Review found that GFR prior to cycle 2 was calculated as 73 mL/min using the Cockroft and Gault formula, the method utilised by the treating institution and thus in accordance with protocol. The Modification of Diet in Renal Disease (MDRD) formula would have given a GFR of 50 mL/min (a decline from baseline), which would have prompted a reduction in vinflunine dose to 280 mg/m^2^ in accordance with trial protocol.

Participant 2: age 70 years, ECOG PS2. He presented on day 14 of cycle 3 with sepsis CTCAE grade 5 that was not associated with neutropenia and that failed to respond to antibiotics and supportive therapy. Three sets of blood cultures failed to identify a causative organism. Review of all previous results indicated persistent grade 1 leucocytosis and intermittent description of fever, raising the suspicion of sub-clinical infection. Two previous serious adverse events were recorded for this patient: bowel obstruction (grade 2) with pyrexia of unknown origin (grade 3) following cycle 1, and pyrexia of unknown origin (grade 3) following cycle 2. A protocol deviation was therefore identified; a dose reduction after cycle 1 did not take place following agreement between the local investigator and senior members of the trial team. IDMC review concluded that failure to reduce the dose had not contributed to this death, since sepsis was not associated with neutropenia.

The toxicity profile of vinflunine was in line with its Summary of Product Characteristics [14]. The most common adverse events of any grade included fatigue (*n* = 17), constipation (*n* = 14), decreased appetite (*n* = 10), and anaemia (*n* = 9) (Table 2). Neutropenia was the most common adverse event of grade ≥3 (*n* = 5) (Table 2 and Supplementary Table 2). Fifteen participants (68%) experienced at least one grade ≥3 adverse event. Supplementary Table 2 shows the distribution of grade ≥3 adverse events by chemotherapy cycle.

**Table 2:** Most frequently reported adverse events (reported by at least two patients).

Specific organ class	Preferred term	Any grade		Grade 3–5	
		*n*	%	*n*	%
Blood and lymphatic system disorders	Anaemia	9	40.9	2	9.1
	Neutropenia	7	31.8	5	22.7
Endocrine disorders	Inappropriate antidiuretic hormone secretion	2	9.1	1	4.5
Gastrointestinal disorders	Abdominal pain	6	27.3	0	0.0
	Aphthous stomatitis	2	9.1	0	0.0
	Constipation	14	63.6	1	4.5
	Diarrhoea	5	22.7	0	0.0
	Flatulence	2	9.1	0	0.0
	Nausea	7	31.8	1	4.5
	Oral pain	4	18.2	1	4.5
	Vomiting	3	13.6	0	0.0
General disorders and administration site conditions	Fatigue	17	77.3	2	9.1
	Influenza-like illness	2	9.1	0	0.0
	Mucosal inflammation	6	27.3	2	9.1
	Oedema peripheral	3	13.6	0	0.0
	Pain	2	9.1	0	0.0
	Pyrexia	5	22.7	2	9.1
Infections and infestations	Rhinitis	2	9.1	0	0.0
	Sepsis	2	9.1	2	9.1
Investigations	Alanine aminotransferase increased	3	13.6	0	0.0
	Weight decreased	5	22.7	0	0.0
	White blood cell count decreased	2	9.1	0	0.0
Metabolism and nutrition disorders	Decreased appetite	10	45.5	0	0.0
	Hyponatremia	2	9.1	2	9.1
Musculoskeletal and connective tissue disorders	Arthralgia	2	9.1	1	4.5
	Back pain	3	13.6	0	0.0
	Groin pain	4	18.2	0	0.0
	Myalgia	2	9.1	0	0.0
	Pain in extremity	3	13.6	1	4.5
	Pain in jaw	2	9.1	0	0.0
Nervous system disorders	Dizziness	3	13.6	0	0.0
	Dysgeusia	5	22.7	0	0.0
	Headache	2	9.1	0	0.0
Psychiatric disorders	Insomnia	7	31.8	0	0.0
Renal and urinary disorders	Renal failure acute	2	9.1	1	4.5
Reproductive system and breast disorders	Penile pain	2	9.1	0	0.0
Respiratory, thoracic and mediastinal disorders	Cough	2	9.1	0	0.0
	Dyspnoea	2	9.1	0	0.0
	Pulmonary embolism	2	9.1	2	9.1
Skin and subcutaneous tissue disorders	Alopecia	6	27.3	0	0.0
	Rash	2	9.1	0	0.0

Percentages calculated in the number of patients who received any vinflunine treatment within the trial. Presented by Medical Dictionary for Regulatory Activity (MedDRA) preferred term, grouped by specific organ class.

## Discussion

This trial has shown that at least 40% of patients with squamous carcinoma of the penis are likely to derive clinical benefit (partial response or disease stabilisation) from vinflunine chemotherapy. CBR (also described as disease stabilisation rate) is usually understood as a composite of rates of objective response (complete and partial) and some measure of non-progression. CBR has not been used as an endpoint in previous penile cancer trials but has shown its value in research scenarios as varied as large-scale randomised studies in non-small cell lung cancer and smaller, all-tumour phase I trials [15, 16]. CBR was chosen as the primary endpoint for VinCaP because objective response rate was felt to be a poor surrogate by which to assess the suitability of vinflunine for further research in our target population. Spontaneous regression and non-progression are not features of metastatic squamous carcinoma of the penis, and stable disease is, therefore, a valid potential marker of activity in a small phase II trial such as this. The choice of CBR also reflects a wider shift in trial endpoints towards measurement of overall disease control (and associated prolongation of survival), such as is seen with the frequent use of progression-free survival in larger studies of anticancer therapy.

Systemic treatment for penis cancer is still dominated by the combination of cisplatin and 5FU, as first described in 1990 [2]. A retrospective review identified a response rate of 32% with overall disease control rate of 72% [4]: the response rate was lower than that first documented, but in keeping with anecdotal experience. The objective response rate for single-agent vinflunine is of a similar magnitude, although this was not the primary endpoint for VinCaP.

The largest prospective clinical trial of chemotherapy in metastatic penis cancer used the combination of bleomycin, methotrexate, and cisplatin in a total of 45 patients [17]. Overall response rate was 32.5% and median overall survival time was 28 weeks, but treatment-related mortality was 13.9%, with 9 patients (20%) withdrawing due to toxicity.

Members of the VinCaP group previously reported on the TPF combination [9]. Objective response rate was 38.5%, with pathological complete remission in 7.6%. This is higher than for vinflunine, but the apparent difference in progression-free survival calls for careful interpretation, given the overlapping 95% CIs (VinCaP 2.9 months, 95% CI: 1.4–6.4 months; TPF 7.1 months, 95% CI: 2.7 to upper limit not reached). TPF also included patients who underwent subsequent surgery with curative intent.

The TIP combination in locally advanced disease [10] has an overall response rate of 50% (pathological complete response in 10%). TIP is clearly the standard of care where down-staging prior to surgery is the objective. Patients with distant metastases are less likely to benefit from such an intensive regimen, as are older patients; median age of patients in VinCaP was 67 years, 10 years older than that of patients in the trial of TIP (median age 57).

Progression-free survival for vinflunine was 2.9 months (95% CI: 1.4–6.4 months), comparable with that of cisplatin–5FU (20 weeks, 95% CI: 11–20 weeks) [4], and cisplatin–methotrexate–bleomycin (14 weeks, 95% CI: 10–21 weeks) [17]. Overall survival in VinCaP seems comparable with these two regimens; the higher overall survival for TIP and TPF may be attributable to the proportion of patients treated in the neoadjuvant setting in those studies. Survival comparisons are summarised in Table 3.

**Table 3:** Summary of outcomes reported for chemotherapy regimens used in the clinical trials for locally advanced or metastatic squamous carcinoma of the penis.

Paper	VinCaP	Pagliaro et al.^10^	Di Lorenzo et al.^4^	Haas et al.^17^	Nicholson et al.^9^
Drugs	Vinflunine	Paclitaxel, cisplatin, ifosfamide	Cisplatin, 5FU	Cisplatin, methotrexate, bleomycin	Docetaxel, cisplatin, 5FU
Trial Population	Metastatic; inoperable locally advanced	Locally advanced	Metastatic; inoperable locally advanced	Locally advanced and metastatic	Locally-advanced and metastatic
PFS/TTP (95% CI)	2.9 months (1.4–6.4)	8.1 months (5.4–50)	20 weeks (11–20)	14 weeks (10–21)	7.1 months (2.7–ULNR)
OS	8.4 months (3.2–14.1)	17.1 months (10.3–>60)	8 months (7–12)	28 weeks (25–35)	13.9 (6.1–ULNR)
ORR	27.3%	50%	32%	32.5%	38.5%
pCR	0	10%	0	12.5%	7.6%

*PFS* progression-free survival, *TTP* time to progression, *CI* confidence interval, *OS* overall survival, *ORR* overall response rate, *pCR* pathological complete remission, *ULNR* upper limit not reached.

There are inevitable limitations to small studies for rarer cancers. Fortunately, the UK system of supranetworks and review of patients in multi-disciplinary team meetings means that no patient who should have undergone down-staging/neoadjuvant TIP chemotherapy would have entered VinCaP. This means that VinCaP participants form a poor prognosis group, reflected in metrics such as response rate and progression-free survival. VinCaP also fails to address the role of vinflunine in patients who relapse after neoadjuvant chemotherapy or as second-line treatment following conventional first-line chemotherapy, where the conclusions of this study may not apply. Our attempt to design a real-world study meant that the method of assessment of renal function was specified as being in accordance with local policy. The inaccuracy of the Cockroft and Gault formula—still in widespread use—has been highlighted elsewhere, and the disparity in GFR calculations for the participant who died with grade 5 acute kidney injury supports the use of either measured GFR or GFR calculated using the MDRD formula prior to each cycle. The two deaths in patients without disease progression are a concern for the potential utility of this agent. Grade 3–5 toxicities, including neutropenia, were documented in one case, indicating that direct treatment toxicity contributed. A causative link is less clear in the second case, given that the patient had sepsis without neutropenia. Haematologic toxicity (including neutropenia) is expected with vinflunine, although grade 3/4 neutropenic infection only affected 6% of pre-treated patients in a large phase III study in urothelial cancer [18]. The small numbers in our study may have over-captured severe toxicity, and vinflunine was otherwise deliverable to this poor prognosis cohort of patients in whom recognised treatment options are limited. There can be no doubt, however, that caution, the use of dose reductions, and the pre-emptive management of constipation are required for vinflunine to be used more widely.

VinCaP was not designed to compare the activity of vinflunine with that of other regimens, but the phase II study of the pan-HER inhibitor dacomitinib [19] merits comment. That study used a single-arm design with a probabilistic statistical model aimed at excluding a response rate of <20%. The study met its primary endpoint. Clinicians treating cancer of the penis now have two new drugs whose activity challenges the long-held hegemony of cisplatin–fluoropyrimidine combinations. There is also interest in exploiting modern immunotherapeutics, with the current PERICLES trial (PEnile Cancer Radio- and Immunotherapy CLinical Exploration Study, NCT03686332), studying atezolizumab with/without concurrent radiotherapy.

Extending research into these very different drugs would require a large-scale randomised trial. The tradition of non-randomised phase II studies in this disease has been overturned with the opening of the International Penile Advanced Cancer Trial (InPACT NCT02305654), which has a complex, multi-arm, multiple-randomisation schema, and aims to recruit 400 patients. That this is feasible is a feature of its Bayesian approach and international collaboration. Bayesian statistics (used in the dacomitinib and TIP trials) eschew hypothesis testing and error minimisation in favour of estimating the probability of identifying the superior treatment. InPACT (recruiting in the UK and the US) provides a template for the design of future trials; a similar international collaboration would rapidly define how best to use these new agents.

### Reporting summary

Further information on research design is available in the Nature Research Reporting Summary linked to this article.

## Supplementary information

Reporting summary checklist

Supplementary table 1

Supplementary table 2

## Data Availability

De-identified data will be made available to other researchers on request, subject to approval of a formal data access request in accordance with the Institute of Cancer Research Clinical Trials and Statistics Unit (IICR-CTSU) data and sample access policy. Trial documentation including the protocol are available on request by contacting vincap-icrctsu@icr.ac.uk. The ICR-CTSU supports the wider dissemination of information from the research it does and increased cooperation between investigators. Trial data are collected, managed, stored, shared, and archived according to ICR-CTSU Standard Operating Procedures in order to ensure the enduring quality, integrity, and utility of the data. Formal requests for data sharing are considered in line with the CR-CTSU procedures with due regard given to funder and sponsor guidelines. Requests are via a standard proforma describing the nature of the proposed research and extent of data requirements. Data recipients are required to enter a formal data sharing agreement, which describes the conditions for release and requirements for data transfer, storage, archiving, publication, and intellectual property. Requests are reviewed by the Trial Management Group (TMG) in terms of scientific merit and ethical considerations including patient consent. Data sharing is allowed if proposed projects have a sound scientific or patient benefit rationale as agreed by the TMG and approved by the Trial Steering Committee as required. Restrictions relating to patient confidentiality and consent will be limited by aggregating and anonymising identifiable patient data. Additionally, all indirect identifiers that might lead to deductive disclosures will be removed in line with Cancer Research UK Data Sharing Guidelines. Additional documents might be shared if approved by the TMG and Trial Steering Committee (e.g. statistical analysis plan and informed consent form).
