# Supplementary material for: VinCaP: a phase II trial of vinflunine in locally advanced and metastatic squamous carcinoma of the penis
Source: Br J Cancer. 2021 Oct 20;126(1):34–41. doi: 10.1038/s41416-021-01574-9 (PMC8727613; doi:10.1038/s41416-021-01574-9)
Supplement: Supplementary file 2 — Supplementary table 1 [file 41416_2021_1574_MOESM2_ESM.docx]

| **Patient** | **Distant mets** | **Local lymph nodes (N stage)** | **Disease in Penis / Penile remnant** | **RECIST assessment** | **Number of cycles received** | **Overall survival (months)** |
| --- | --- | --- | --- | --- | --- | --- |
| Locally-Advanced Disease only | | | | | | |
| 1 | 0 | 0 | Yes | SD | 5 | 8.4 |
| 2 | 0 | 2 | No | PR | 4 | Censored (27.9) |
| 3 | 0 | 3 | No | PR | 8 | 21.3 |
| 4 | 0 | 3 | No | Not evaluable | 0 | 0.6 |
| 5 | 0 | 3 | No | CR | 7 | Censored (14.1) |
| 6 | 0 | 3 | No | SD | 6 | Censored (16.1) |
| 7 | 0 | 3 | Yes | Discontinued (AE) prior to RECIST | 1 | 3.7 |
| 8 | 0 | 3 | Yes | PR | 7 | Censored (14.1) |
| 9 | 0 | 3 | Yes | Discontinued (AE) prior to RECIST | 1 | 21.5 |
| 10 | Penile shaft | 3 | Yes | Discontinued (AE) prior to RECIST | 1 | 10.2 |
| Distant Metastatic Disease | | | | | | |
| 11 | Lung | 0 | No | Not evaluable | 0 | Censored (0.0) |
| 12 | Lung, LN | 0 | No | CR | 6 | Censored (4.5) |
| 13 | Lung, Spleen | 0 | No | SD | 4 | 10.2 |
| 14 | Perineum | 0 | No | SD | 4 | Censored (7.3) |
| 15 | Pleura, Heart | 2 | No | Died prior to RECIST | 3 | 2.1 |
| 16 | Skin | 2 | No | PD | 2 | 2.0 |
| 17 | Lung | 2 | No | PD | 2 | 2.9 |
| 18 | Lung, LN | 3 | No | PR | 8 | 11.7 |
| 19 | Skin | 3 | No | PD | 1 | 3.2 |
| 20 | Lung, Bone, Bladder | 3 | No | PD | 3 | 5.0 |
| 21 | Liver, LN | 3 | No | Not evaluable | 0 | 0.8 |
| 22 | Lung | 3 | No | PD | 4 | 5.4 |
| 23 | Lung | 3 | No | PD | 4 | 8.4 |
| 24 | Liver | 3 | Yes | PD | 2 | 14.1 |
| 25 | LN | 3 | Yes | Died prior to RECIST | 2 | 1.1 |

**Supplementary table 1.** RECIST response and overall survival in relation to patient characteristics.
