# Supplementary material for: VinCaP: a phase II trial of vinflunine in locally advanced and metastatic squamous carcinoma of the penis
Source: Br J Cancer. 2021 Oct 20;126(1):34–41. doi: 10.1038/s41416-021-01574-9 (PMC8727613; doi:10.1038/s41416-021-01574-9)
Supplement: Supplementary file 3 — Supplementary table 2 [file 41416_2021_1574_MOESM3_ESM.docx]

| **Adverse event** | **Grade** | **Chemotherapy cycle** | | | | |
| --- | --- | --- | --- | --- | --- | --- |
|  |  | **1** | **2** | **3** | **4** | **7** |
| Anaemia | 3 | 0 | 2 | 0 | 0 | 0 |
| Arthralgia | 3 | 0 | 1 | 0 | 0 | 0 |
| Constipation | 3 | 1 | 0 | 0 | 0 | 0 |
| Fatigue | 3 | 1 | 1 | 0 | 0 | 0 |
| Febrile neutropenia | 3 | 0 | 0 | 1 | 0 | 0 |
| Hypercalcemia | 3 | 1 | 0 | 0 | 0 | 0 |
| Hyponatremia | 3 | 0 | 0 | 0 | 0 | 1 |
|  | 4 | 0 | 0 | 1 | 0 | 0 |
| Inappropriate antidiuretic hormone secretion | 3 | 1 | 0 | 0 | 0 | 0 |
| Infection | 3 | 0 | 0 | 0 | 1 | 0 |
| Mucosal inflammation | 3 | 0 | 0 | 1 | 0 | 1 |
| Muscular weakness | 3 | 0 | 1 | 0 | 0 | 0 |
| Nausea | 3 | 0 | 1 | 0 | 0 | 0 |
| Neutropenia | 3 | 1 | 1 | 1 | 0 | 0 |
|  | 4 | 1 | 1 | 0 | 0 | 0 |
| Neutropenic sepsis | 5 | 0 | 1 | 0 | 0 | 0 |
| Oral pain | 3 | 0 | 0 | 1 | 0 | 0 |
| Pain in extremity | 3 | 1 | 0 | 0 | 0 | 0 |
| Pelvic pain | 3 | 0 | 0 | 1 | 0 | 0 |
| Pulmonary embolism | 3 | 1 | 0 | 0 | 1 | 0 |
| Pyrexia | 3 | 2 | 0 | 0 | 0 | 0 |
| Renal failure acute | 5 | 0 | 1 | 0 | 0 | 0 |
| Sepsis | 4 | 0 | 1 | 0 | 0 | 0 |
|  | 5 | 0 | 0 | 1 | 0 | 0 |
| Tumour pain | 3 | 0 | 1 | 0 | 0 | 0 |
| Urinary tract infection | 3 | 0 | 0 | 1 | 0 | 0 |

**Supplementary table 2.** Number of patients reporting grade ≥3 adverse events by chemotherapy cycle. Presented by Medical Dictionary for Regulatory Activity (MedDRA) preferred term, grouped by specific organ class. The grade 5 events of neutropenic sepsis and renal failure acute were reported by the same patient. No grade 3-5 adverse events were reported in cycles 5-6 or 8.
